# Modeling the development of proteolytic phenotypes in multi-species oral biofilms

**DOI:** 10.1080/20002297.2017.1325274

**Published:** 2017-06-09

**Authors:** Julia R. Davies, Bertil Kinnby, Gunnel Svensäter

**Affiliations:** ^a^ Department of Oral Biology, Malmö University, Malmö, Sweden

## Abstract

In chronic periodontitis, subgingival biofilms induce an inflammatory response leading to periodontal tissue destruction which may cause tooth loss. The response includes exudation of fluid (GCF) from the gingival pocket, giving rise to a protein-rich micro-environment in the biofilm. We hypothesize that proteolytic activity in biofilms is a virulence factor contributing to sustained inflammation.

To study the influence of GCF on proteolytic activity in a periodontitis-associated biofilm, a multi-species consortium (*Porphyromonas gingivalis, Fusobacterium nucleatum, Parvimonas micra* and *Streptococcus constellatus*) was grown in 10% equine serum (to model GCF) or BHI (control) for 2-5 days. Cell-associated and secreted proteolytic activity was investigated with zymography, fluorometry or fluorescent substrates in combination with fluorescence *in situ* hybridization (FISH).

After 2 days, streptococci dominated the consortium in BHI whereas in serum diversity was maintained over 5 days. The serum consortium also developed proteolytic activity, which was absent in BHI. Zymography revealed an array of secreted proteases. FISH revealed proteolytic activity associated with the cell surface of *P. gingivalis* and *F. nucleatum* but not *P. micra* or *S. constellatus*.

Thus, serum favored survival of *P. gingivalis* and *F. nucleatum* as well as production of proteases that can act as virulence factors in chronic periodontitis.

